# Long-Term Impact of Single Epilepsy Training on Knowledge, Attitude and Practices: Comparison of Trained and Untrained Rwandan Community Health Workers

**DOI:** 10.3389/ijph.2021.645598

**Published:** 2021-11-18

**Authors:** Peter Dedeken, Stephen N. Muhumuza, Fidele Sebera, Josiane Umwiringirwa, Leopold Bitunguhari, Hans Tierens, Dirk E. Teuwen, Paul A. J. M. Boon

**Affiliations:** ^1^ Department of Neurology, Ghent University Hospital, Ghent, Belgium; ^2^ Department of Neurology, Heilig Hart Ziekenhuis, Lier, Belgium; ^3^ UCB Pharma, Brussels, Belgium; ^4^ School of Medicine and Pharmacy, University of Rwanda, Kigali, Rwanda; ^5^ Kabgayi District Hospital, Kabgayi, Rwanda; ^6^ CARAES Neuro-psychiatric Hospital, Brothers of Charity, Department of Neurology, Kigali, Rwanda; ^7^ Centre Hospitalier Universitaire (CHU-K), Kigali, Rwanda; ^8^ Dataroots BV, Leuven, Belgium

**Keywords:** epilepsy, knowledge, attitude, practices, community health workers, training

## Abstract

**Objectives:** To close the epilepsy treatment gap and reduce related stigma, eradication of misconceptions is importantIn 2014, Community Health Workers (CHWs) from Musanze (Northern Rwanda) were trained on different aspects of epilepsy. This study compared knowledge, attitude and practices (KAPs) towards epilepsy of trained CHWs 3 years after training, to untrained CHWs from Rwamagana (Eastern Rwanda).

**Methods:** An epilepsy KAP questionnaire was administered to 96 trained and 103 untrained CHWs. Demographic and intergroup KAP differences were analysed by response frequencies. A multivariate analyses was performed based on desired and undesired response categories.

**Results:** Epilepsy awareness was high in both groups, with better knowledge levels in trained CHWs. Negative attitudes were lowest in trained CHWs, yet 17% still reported misconceptions. Multivariate analysis demonstrated the impact of the training, irrespective of age, gender and educational level. Knowing someone with epilepsy significantly induced more desired attitudes.

**Conclusion:** Despite demographic differences between trained and untrained CHWs, a single epilepsy training resulted in significant improvement of desired KAPs after 3 years. Nation-wide CHW training programs with focus on training-resistant items, e.g., attitudes, are recommended.

## Introduction

Epilepsy is a common chronic neurological disorder affecting people of all ages. Up to 80% of the 70 million people living with epilepsy (PwE) in the world live in low- and middle-income countries [1].

In sub-Saharan Africa (SSA), epilepsy is associated with a high “triple” burden of disease. High prevalence is the first burden, often secondary to treatable or avoidable causes such as infectious diseases, limited perinatal care, and traumatic brain injury [2–6]. In a meta-analysis of SSA community-based door-to-door surveys the prevalence of active epilepsy is estimated at 9.0‰ and lifetime epilepsy at 16‰, which is much higher compared to High Income Countries or Latin America and Asia [7]. In Rwanda, prevalence of 49‰ reported in 2005 greatly exceeds the SSA prevalence and was recently re-confirmed by a door-to-door survey in three villages of Musanze district in the Northern province in 2017 [5].

A second burden is the epilepsy diagnosis gap and epilepsy treatment gap, due to a healthcare infrastructure with limited resources and inadequate access to antiepileptic drugs. Closing the epilepsy diagnosis gap is hampered by a limited number of neurologists, epilepsy-trained staff and lack of access to EEG investigations and imaging [8]. The epilepsy treatment gap in SSA is estimated at 68.5% (95% CI: 59.5–77.5%), double in rural compared with urban regions [9, 10]. The epilepsy treatment gap in Rwanda in 2005 was 67.8% [5].

The third burden relates to various forms of social discrimination and stigma, even more important in vulnerable groups, such as women and children in remote areas. In several African communities the myths and beliefs regarding epilepsy impact the epilepsy treatment gap [11–14]. Misconceptions about epilepsy result in negative social, psychological and economic consequences, such as fear, humiliation, social and work exclusion [12, 15–19].

To overcome these barriers, CHWs may play a key role in mobilization of patients, referral to primary healthcare facilities and provide education. Engagement programs with CHWs in Rwanda have been critical in turning around the burden of malaria and maternal death, demonstrating CHW’s undisputed value [20, 21].

As CHWs are recruited amongst villagers, there is a need to confirm that misconceptions and beliefs have been altered and, more importantly, persist on the long term after training on epilepsy. The Rwandan Organisation Against Epilepsy (ROAE), a chapter of the International League Against Epilepsy (ILAE), engaged with Community Health Workers (CHW) to increase epilepsy diagnosis and referral at the grassroot community level by providing epilepsy training courses in the Northern Province. This article discusses difference in knowledge, attitude and perception (KAP) parameters in CHWs trained by the ROAE compared to CHW not having been exposed to an epilepsy training, at least 3 years after the initial training.

## Methods

### Study Setup

This cross-sectional study was conducted in a cluster of semi-urban and rural villages in Musanze district, Northern Province, where the ROAE had performed training courses to CHWs. A cluster of villages in the eastern rovince from the Nyakariro sector, Rwamagana district, Eastern Province, was selected as a control area as no known epilepsy training or awareness programs had been conducted before by any non-governmental organisation or mental health program In Rwanda, each village is covered by 3 or 4 CHWs, each having their respective assignments.

The study was approved by the ethical committee of the University Hospital of Kigali, CHUK. Participants provided written informed consent. A financial compensation of 2000 RwFr (2.33USD, Feb 2018 exchange rate) was provided in line with the recommendation of the Rwandan Government [22].

### Study Population and Exclusion Criteria

We recruited epilepsy trained CHWs in the Northern Province (group A) and untrained CHWs from the Eastern Province (group B).

CHWs in Group A were enrolled if they had attended the 2014 ROAE epilepsy training. Group B consisted of CHWs from the Nyakariro sector who had never attended an epilepsy training. CHWs were identified by their supervisor and invited to a meeting in their village. Participants were excluded if they were illiterate or had relocated since 2014. All were native Kinyarwanda speaking.

### Epilepsy Training

In 2014, 2,472 CHWs in the Musanze District received, between June and November 2014, an epilepsy training by the ROAE, organised in collaboration with and under supervision of local health authorities. Training elements included symptoms of epilepsy, including video cases, causes, treatment, prognosis, social aspects, etc., (Table 1) The content had been adapted to the schooling level of CHWs, of whom some are illiterate.

**TABLE 1:** Training topics. Rwandan Organisation against Epilepsy training program of Community Health Worker epilepsy, Rwanda, 2014.

	Topic
1	History of epilepsy
2	Epidemiology of epilepsy
3	Socio-cultural perception, including community rejection of PwE
4	Definition and anatomy and neurophysiological aspects of epilepsy
5	Aetiology
6	Diagnosis
7	Risk factors and triggers
8	Treatment
9	Taking charge during an epileptic seizure
10	Epilepsy in everyday life
11	Clinical cases on video

### Questionnaire

A questionnaire was developed by the team, based on different published data [3, 23, 24]. After selection of questions, it was translated into Kinyarwanda. The translation was adapted to address cultural and linguistic aspects and the final version was validated by a neurologist. The self-administered questionnaire contained 4 questions on demographics and 14 questions on epilepsy knowledge/awareness, attitude and practice. We anticipated in the untrained group that CHWs would be unfamiliar with epilepsy and allowed for every question the option “not familiar”. The principal investigator and three nurses assisted during the questionnaire administration to CHWs in groups by village or by district.

### Sample Size and Data Collection

This was an explorative study as long-term retention of KAPs by CHWs, to our knowledge, has not yet been assessed. We aimed for enrolment of 100 CHW in each group following a feasibility assessment that the project would involve nearly 30 villages for each group. Also, as CHWs might have relocated, we anticipated a difficult recruitment of previously trained CHWs.

Data collection was performed in January 2018 and March 2018.

### Statistical Analysis

Double data entry into Google Forms was completed by an independent data specialist and data were extracted to Microsoft Excel. Statistical analysis, using STATA 12 software, was descriptive. Intergroup differences were calculated using two tailed Z-test or t-test as appropriate for continuous variables and Chi^2^-comparison for categorical variables using Yates correction where applicable.

To explore the effect of training on long-term KAP change in CHWs, multiple count regressions were performed using the R software.

First, we transformed our results to “desired” and “undesired” responses by question and by subdimension score (knowledge, attitude and practice) as per Table 3. Then we computed our results by counting the number of desired versus undesired answers for each question, correcting for undesired answers in multiple entry fields. We compiled a subdimension score by summing up the number of questions with a desired response and generated an overall score by summing all sub scores. The response option “not familiar with epilepsy” was considered as undesirable across all subdomains. Lastly, we fitted the multiple count regressions for each subdimension.

Missing answers were accounted for by controlling for the number of answered questions. We also performed a sensitivity analysis in which we considered missing values as undesired answers using the same model.

## Results

### Demographic Characteristics

A total of 199 CHWs were recruited, with 99 group A CHWs from 33 villages in the Muhoza, Cyuve and Musanze sectors, and 100 group B CHWs from 38 villages in the Nyakiriro sector. Upon data analysis and reconciliation with training records from 2014, three group A CHWs had not attended any epilepsy training and therefore were analysed in group B as untrained (Table 2).

**TABLE 2:** Demographics of Community Health Workers. Survey on epilepsy Knowledge, Attitudes and Practices in Community Health Workers, Rwanda, 2018.

	Group A	Group B
Gender, N	96	103
Female, n (%)	66 (68.8)	70 (68.0)
	*p* > 0.05	
Age, N	96	103
Mean	45.0	40.0
Median (y)	45.2	40.1
Minimum–Maximum (y)	24–86	25–65
	*p* < 0.001	
Urban vs. rural, N	96	102^a^
Urban, n (%)	83 (86.5)	3 (2.9)
Rural, n (%)	13 (13.5)	99 (97.1)
	*p* < 0.001	
Education level, N	96	102^a^
Primary school, n (%)	42 (43.8)	81 (78.6)
Secondary school, n (%)	45 (46.9)	14 (13.7)
Vocational level, n (%)	9 (9.4)	7 (6.9)
	*p* < 0.001	

a Data missing for one Community Health Worker.

CHWs were predominantly female and the trained group had a significantly higher proportion of older participants. In line with a more frequently urban provenance in group A, in contrast to rural provenance in group B, we also observed higher schooling level in this group.

### Awareness of Epilepsy

Most participants had heard of epilepsy, yet significantly different favouring the trained group, with only one CHW in the trained group A not able to recall epilepsy as a disease, 3 years after training (Table 3).

**TABLE 3:** Awareness of epilepsy. Survey on epilepsy Knowledge, Attitudes and Practices in Community Health Workers, Rwanda, 2018.

	Group A (N = 96)	Group B (N = 103)
	n (%)	n (%)
Do you know a person with epilepsy?	N = 95	N = 103
Yes	81 (85.3)	72 (73.5)
No	13 (13.7)	26 (26.5)
Not familiar	1 (1.1)	5 (4.7)
	*p =* 0.14	
Have you ever witnessed seizure?	N = 96	N = 103
Yes	93 (96.3)	89 (86.4)
No	3 (3.10)	9 (8.7)
Not familiar	0 (0.0)	5 (4.9)
	*p=* 0.075	
If you witnessed a seizure, what number of symptoms did you observe^a^	N = 93	N = 89
1 symptom	12 (12.9)	4 (4.5)
2 symptoms	19 (20.4)	6 (6.7)
3 symptoms	22 (23.7)	18 (17.5)
4 symptoms	28 (30.1)	16 (15.5)
5 or more symptoms	12 (12.9)	45 (50.6)
	*p <* 0,001	

a Results reworked from original question: if you observed a seizure which of the following symptoms did you observe? Options included confusion, tongue bite, loss of urine, coma/loss of consciousness, stiffness and eye deviation.

Of interest is that over 85% of untrained CHWs had witnessed a seizure indicating good familiarity with seizures or patients living with epilepsy (PwE). This rate was similar in both groups.

### Knowledge and Attitudes Towards Epilepsy Among CHWs

Epilepsy knowledge was better in trained CHWs, providing more correct answers on the cause of epilepsy. Epilepsy was recognised by more than 80% in group A and 60% in group B as a brain disease, yet epilepsy as madness or spiritual possession was reported by nearly 1 in 6 of trained CHWs.

Overall, trained CHWs differ in attitudes towards PwE compared to untrained CHWs, both on items related to personal avoidance and fear as well as negative stereotypes, work and role expectations (Table 4). Nearly 1 in 5 trained CHWs, however, considered epilepsy a possibly contagious disease. Varying response rates with more missing values were observed in group B. Between 1 and 20 untrained CHWs choose to respond “not familiar with epilepsy” to different questions. Interestingly in the untrained group, response rates regarding attitudes were higher than on awareness or knowledge.

**TABLE 4:** Attitudes, knowledge and practices towards epilepsy. Survey on epilepsy Knowledge, Attitudes and Practices in Community Health Workers, Rwanda, 2018.

	Group A (N = 96)	Group B (N = 103)
	n (%)	n (%)
KNOWLEDGE		
**Have you heard/read about epilepsy?**	N = 96	N = 103
Yes (D)	95 (99.0)	91 (88.3)
No (UD)	1 (1.0)	12 (11.7)
	*p* = 0.006	
**Which age group is most affected** **?** ^**a**^	94 respondents	101 respondents
	N = 95	N = 109
0–4 (UD)	7 (7.4)	27 (24.8)
5–9 (UD)	21 (22.1)	34 (31.2)
≥10 (D)	67 (70.5)	48 (44.0)
	*p* < 0.001	
**What is the cause of epilepsy?** ^**a**^	95 respondents	82 respondents
	N = 206	N = 156
Brain injury (D)	86 (41,7)	61 (39.1)
Genetic trait (D)	14 (6.8)	21 (13.5)
Birth injury (D)	59 (28.6)	13 (8.3)
Excessive worry (UD)	27 (13.1)	15 (9.6)
Blood disorders (D)	17 (8.3)	17 (10.9)
Witchcraft (UD)	1 (0.5)	1 (0.8)
Curse of God (UD)	0 (0.0)	0 (0.0)
Spirit possession (UD)	1 (0.5)	8 (5.1)
Not familiar (UD)	1 (0.5)	20 (12.8)
	*p* < 0.001	
**Is epilepsy… ^a^**	96 respondents	89 respondents
	N = 114	N = 105
Madness (UD)	6 (5.3)	9 (7.6)
Spirit possession (UD)	12 (10.5)	13 (11.0)
Mental retardation (UD)	3 (2.6)	5 (4.2)
Brain disease (D)	93 (81.6)	78 (66.1)
Not familiar (UD)	0 (0.0)	13 (11.0)
	*p* = 0.013	
ATTITUDES		
**Would you allow your child to play with a child living with epilepsy?**	N = 96	N = 103
Yes (D)	93 (96.9)	84 (81.6)
No (UD)	3 (3.1)	7 (6.8)
Not familiar (UD)	0	12 (11.7)
	*p* = 0.003	
**A child living with epilepsy should never attend school?**	N = 96	N = 102
True (UD)	11 (11.5)	16 (15.7)
False (D)	80 (83.3)	70 (68.6)
Sometimes true (UD)	5 (5.2)	4 (3.9)
Not familiar (UD)	0 (0.0)	12 (11.8)
	*p* = 0.011	
**May your son marry a woman living with epilepsy?**	N = 95	N = 102
Yes (D)	80 (84.2)	51 (50.0)
No (UD)	15 (15.8)	38 (37.3)
Not familiar (UD)	0 (0.0)	13 (12.7)
	*p* < 0.001	
**May your daughter marry a man living with epilepsy?**	N = 96	N = 103
Yes (D)	84 (87.5)	52 (50.5)
No (UD)	12 (12.5)	40 (38.8)
Not familiar (UD)	0 (0.0)	11 (10.7)
	*p* < 0.001	
**Can you give a job to a person living with epilepsy?**	N = 96	N = 102
Yes (D)	91 (94.8)	68 (66.7)
No (UD)	5 (5.2)	34 (33.3)
Not familiar (UD)	0 (0.0)	0 (0.0)
	*p* < 0.001	
**Is epilepsy is contagious?**	N = 94	N = 100
Always (UD)	1 (1.1)	7 (7.0)
Sometimes (UD)	16 (17.0)	29 (29.0)
Never (D)	77 (81.9)	64 (64.0)
Not familiar (UD)	0 (0.0)	0 (0.0)
	*p* = 0.026	
PRACTICES		
**If a person found on the road what would you do?^a^**	96 respondents	97 respondents
	N = 96	N = 110
Health facility (D)	95 (99.0)	84 (76.4)
Traditional healer (UD)	0 (0.0)	3 (2.7)
Chinese medicine (UD)	0 (0.0)	3 (2.7)
Praying session (UD)	0 (0.0)	5 (4.5)
Do nothing as there is no treatment (UD)	0 (0.0)	5 (4.5)
Do not know (UD)	1 (1.0)	4 (3.6)
Not familiar (UD)	0 (0.0)	6 (5.5)
	*p* = 0.025	
**What would you recommend for treatment for a family member living with epilepsy?^a^**	96 respondents	98 respondents
	N = 106	N = 121
Health facility (D)	96 (90.6)	92 (76.0)
Traditional healer (UD)	0 (0.0)	8 (6.6)
Chinese medicine (UD)	2 (1.9)	3 (2.5)
Prayers (UD)	8 (7.5)	11 (9.1)
No, as epilepsy cannot be treated (UD)	0 (0.0)	2 (1.7)
Do not know what to do (UD)	0 (0.0)	1 (0.8)
Not familiar (UD)	0 (0.0)	4 (3.3)
	*p* = 0.18	
**How long should the treatment be given?^b^**	94 respondents	102 respondents
	N = 98	N = 102
For 1 week (UD)	3 (3.1)	11 (10.8)
For 1 year (UD)	5 (5.1)	7 (6.9)
For 2 years of seizure freedom (D)	45 (45.9)	17 (16.7)
For life (D)	45 (45.9)	67 (65.6)
	*p* < 0.001	

^a^ More than one answer was allowed.

^b^ Some Community Health Workers selected two answers with n, number responses; D, desired response; UD, undesired response.

### Treatment Practices by CHWs

Group A CHWs were more likely to send suspect cases of epilepsy to health centres for diagnosis and treatment (Table 3) with 99% of trained CHWs referring to medical facilities both relatives and unrelated persons. In both groups, there was a tendency to seek alternative care by traditional or faith healers when referring a relative.

### Training Effect and Multivariate Analysis

The use of regression analysis allowed taking advantage of cross-sectional data, by controlling for confounding factors such as gender, age, education and epilepsy awareness, such as knowing someone with epilepsy.

The analysis demonstrated statistically significant effects of the training on awareness and knowledge, attitude and overall score, but not on treatment practice (Table 5). No control variables showed statistical effects, except for epilepsy awareness that impacted the attitude dimension. In adidtion, previous exposure by knowing someone with epilepsy was associated with a better score on the attitude questions. Educational level seemed to trend for higher impact on across all subdimensions by higher compared to lower educational levels, yet this effect was small and not significant. As a missing result could have been intentional and therefore could have been classified as an undesired answer, a sensitivity analysis was conducted considering missing answers as undesired. Results from our initial analysis were confirmed.

**TABLE 5:** Multivariate analysis of training effect and confounding factors. Survey on epilepsy Knowledge, Attitudes and Practices in Community Health Workers, Rwanda, 2018.

Effect of Interest	Knowledge	Attitude	Practice	Overall
	B (SE)	B (SE)	B (SE)	
Training	**0.217 (0.091)**	**0.286 (0.076)**	0.110 (0.098)	**0.219 (0.050)**
*p*-value	**0.018**	**<0.001**	0.263	**<0.001**
Control Variables				
Know Someone with Epilepsy	0.019 (0.101)	**0.196 (0.089)**	0.004 (0.108)	0.089 (0.057)
*p*-value	0.850	**0.027**	0.970	0.115
Gender (ref. female)	0.045 (0.089)	0.062 (0.074)	0.061 (0.095)	0.057 (0.049)
*p*-value	0.613	0.402	0.521	0.243
Age	−0.003 (0.004)	−0.0001 (0.004)	0.001 (0.005)	−0.001 (0.002)
*p*-value	0.504	0.723	0.841	0.660
Education Secondary (ref. Primary)	0.071 (0.096)	0.042 (0.079)	0.055 (0.103)	0.054 (0.053)
*p*-value	0.463	0.592	0.594	0.303
Education Vocational (ref. Primary)	0.072 (0.148)	−0.043 (0.127)	0.003 (0.163)	0.005 (0.083)
*p*-value	0.626	0.738	0.985	0.955
Intercept	−2.900 (0.196)	−2.762 (0.163)	−3.145 (0.208)	−1.826 (0.107)
*p*-value	<0.001	<0.001	<0.001	<0.001

B are regression coefficients on the natural log-scale. SE, standard error. The response was scaled by the number of completed answers for each (sub)dimension. Statistically significant results, based on two-sided *p*-values and on the 95% confidence interval, are reported in bold.

## Discussion

In Rwanda, CHWs are key grassroot health contributors on public health in their communities and have been instrumental in improving perinatal care and the combat against malaria. To change misconceptions and beliefs on epilepsy in the community as well as to increase detection and referral of possible PwE, CHWs were trained on epilepsy in a selected region in Rwanda. Three years after the initial single training session, we compared KAPs from trained and untrained CHWs, to assess training gaps and needs for repeat training programs.

According to our multivariate analysis, the training effect is the only observed variable that explains the difference in results between trained and untrained CHWs in terms of knowledge, attitudes, practices and overall KAP score, despite significant demographic differences between our samples. However, we could not control for the effect of provenance (rural vs. semi-urban) because of the imbalance of subjects in our dataset (97% of the untrained subjects were living in rural villages). This imbalance between samples was unexpected, as the overall profile of the Musanze and Rwamagana health districts are very similar and despite a large recruitment area of more than 30 villages. Group A was predominantly from an urban setting, which may also explain the significant difference found on schooling. Educational level alone cannot sufficiently explain our results as it accounted for only a small and non-significant effect on attitudes, practices and overall score. Interestingly, higher age may have negative contribution on desired KAPs which may be interpreted that KAPs are may be more difficult to change in older CHWs, yet this result requires cautious interpretation and further investigation.

Epilepsy awareness was high in both groups, with only 1 in 9 untrained CHWs not having heard of epilepsy. In addition, knowing someone with epilepsy positively and significantly impacted attitudes towards epilepsy. Therefore, bringing PwE closer to CHWs may also be considered a strategy to decrease negative attitudes.

Trained CHWs retained good knowledge and were able to detail main causes of epilepsy, signs and symptoms and treatment. Untrained CHWs listed often five or six symptoms observed during a seizure, which may in correspond to recognition of tonic-clonic seizures.

The results of trained CHWs in the attitude towards epilepsy were encouraging with a significant difference compared to untrained CHWs on aspects of personal fear and social exclusion as well as work/role expectations. This lasting effect of the training course and understanding of the disease burden was remarkable. Yet, still one in 10 trained CHWs would exclude children with epilepsy from schooling. Moreover, up to 15% of trained and 20% of untrained CHWs considered epilepsy as a curse or madness. A single training did not eradicate some beliefs and likely that socio-cultural and traditional beliefs persisted [25, 26]. Indeed, trained CHWs still reported epilepsy as possibly contagious in 17.7%, compared to untrained CHWs in 35.0%. This has been equally illustrated in other countries [11, 19, 25, 26].

Epilepsy management choices were well retained in trained CHWs, but also up to 80% of untrained CHWs would refer to the health facilities. Interestingly, the referral of a relative in group A and B involved also more frequently traditional healer referral, with prayers as an important pathway, as illustrated in Uganda [27]. These referral patterns may prove very important for treatment seeking patterns in patients and require utmost attention [28].

In general, there was more uncertainty in untrained CHWs with more responding “not familiar with epilepsy” in epilepsy knowledge and management questions. Interestingly, for untrained CHWs, more responses on questions regarding attitudes were recorded than on knowledge. We hypothesize that in community’s epilepsy as a disease is surrounded by socio-cultural beliefs and misconceptions, demonstrating the knowledge gap.

### Epilepsy Training Course Opportunities

Addressing changing attitudes towards epilepsy will be important as some misconceptions persisted in a significant proportion of trained CHWs, e.g., nearly 1 in 5 trained CHW reported epilepsy still as a contagious disease. Future programs and training materials need to emphasize and elaborate on those identified training-resistant topics, mainly related to attitudes, personal vs. societal beliefs and cultural aspects. These future programs should equally address vulnerable persons living with non-communicable diseases in the local communities, including women and children. Considering CHWs are best placed to support referral of and adherence to treatment, including the use of anti-epileptic drugs in PwE, the removal of misconceptions is crucial [3, 29].

Addressing an active engagement of CHW will also be important. In line with African culture, verbally transmitted information seems more impactful than written information [30]. CHWs are respected advocates and influencers in their local communities. It would be interesting to assess changes of KAPs on epilepsy or any condition in the lay population after a disease specific training program. This would, of course, require a scaling of testing at different time points with validated scales, sensitive to changes over time and to geographical and cultural differences.

The contribution of CHW in reducing the epilepsy diagnosis gap and epilepsy treatment gap has been acknowledged in other SSA countries [31–33]. The ROAE training program in 2014 had two objectives: 1) increase the disease knowledge of CHW; and, 2) improve the referral of PwE to local healthcare centres (Table 5). The impact of the CHW training in the referral of PwE to healthcare centres was sub-optimal. The number of PwE seen at the healthcare centres was much lower than anticipated on the reported prevalence of 49‰ for Rwanda [5]. The role and impact of CHW increases if properly equipped with adequate educational tools and the use of simple questions on screening for epilepsy proved efficient [29, 30].

Although clear needs for repeat training programs were identified with persisting negative attitudes and knowledge gap, public health managers may consider initially to scale single training of CHWs on epilepsy nationwide and include more specific attitude, belief and behaviour training topics. Such single training at large scale may be more impactful than conducting repeat training programs in lower numbers of CHWs.

In addition, we believe that future epilepsy training projects should be expanded towards other influencers in their communities, such as, traditional healers, schoolteacher and trainers at sports clubs. Indeed, inclusion of community influencers may increase the impact of epilepsy training course as they may be close to children and adolescents, representing a vulnerable population [12]. Also, traditional or faith healers may contribute the closure of the diagnosis gap and treatment gap. Healthcare seeking behaviour is complex and may be driven by erroneous beliefs, attitudes and limited social support [34]. In our study referral was geared to biomedical care although often recommendations for traditional healers would be made, even more so to relatives of the CHWs. Alternative care offered by traditional or faith healers, especially in rural areas, may delay the biomedical healthcare seeking behaviour. In acute paediatric conditions in Rwanda, the use of traditional healers was the most significant predictor of the delay [35]. An epilepsy training for these alternative healthcare providers, trusted and influencers in their communities, seems complementary and recommended to a CHW training [3]. Even more, involving all stakeholders across biomedical care, i.e*.*, physicians, nurses and social workers, and CHWs, on one hand and traditional healers, on the other hand, may even increase impact by fostering mutual understanding, interaction, collaboration and creation of win-win contribution to holistic care [12, 36].

In view of the demonstrated benefits of the CHWs epilepsy training and of their closeness and social acceptability to villagers, nationwide training programs with adequate tools, will further advance epilepsy care, reduce stigma, improve social integration at work and at schools in Rwanda, addressing an important burden given the high prevalence of epilepsy [5].

### Study Limitations

An important limitation relates to the absence of a pre-training and immediate post-training assessment of KAPs in the trained CHWs; hence, we could not assess a change vs. pre-training KAPs nor decay thereof over time. We recommend that future training programs use evaluation tools pre- and immediately post-training to track time sensitive changes of the impact of an intervention.

Another limitation is the use of a non-validated questionnaire. The instrument was a Kinyarwanda translation based on selected elements from other questionnaires [3, 23, 24]. This may have induced unidentified gaps and untested items. Translation and validation of specific instruments in Kinyarwanda, such as the Stigma Scale in Epilepsy is recommended [3]. Third, we excluded illiterate CHWs from the study, which may have favored the training effect. On the other hand, education level did only have a non-significant trend towards more desired responses for higher educational levels.

### Conclusion

Despite demographic differences between trained and untrained CHWs, our data suggest that even after 3 years, differences in KAPs were driven by a single epilepsy training.

Further assessments of the impact of the training and the outcome for the PwE are recommended and easy to administer validated questionnaires should be applied, both pre- and post-training. A nation-wide training programs of CHWs with targeted information modules on epilepsy, focused on specific attitude, beliefs and practices, should be recommended. In addition to training, equipping CHWs with easy-to-use tools, such as screening questions and treatment guidance, could drive closure of the epilepsy diagnosis and treatment gap. Indeed, well-trained CHWs will contribute to change negative attitudes and beliefs toward epilepsy and promote positive behaviour toward PwE. With their support, PwE are likely to face less stigma and discrimination, to better access neurological care and to become better integrated in their communities.

## Data Availability

Anonymized study data will be accessible upon request to the corresponding author and team approval, within a reasonable timeframe.
