# Corrigendum: Long-Term Impact of Single Epilepsy Training on Knowledge, Attitude and Practices: Comparison of Trained and Untrained Rwandan Community Health Workers

**DOI:** 10.3389/ijph.2023.1606486

**Published:** 2023-11-06

**Authors:** Peter Dedeken, Stephen N. Muhumuza, Fidele Sebera, Josiane Umwiringirwa, Leopold Bitunguhari, Hans Tierens, Dirk E. Teuwen, Paul A. J. M. Boon

**Affiliations:** ^1^ Department of Neurology, Ghent University Hospital, Ghent, Belgium; ^2^ Department of Neurology, Heilig Hart Ziekenhuis, Lier, Belgium; ^3^ UCB Pharma, Brussels, Belgium; ^4^ School of Medicine and Pharmacy, University of Rwanda, Kigali, Rwanda; ^5^ Kabgayi District Hospital, Kabgayi, Rwanda; ^6^ CARAES Neuro-psychiatric Hospital, Brothers of Charity, Department of Neurology, Kigali, Rwanda; ^7^ Centre Hospitalier Universitaire (CHU-K), Kigali, Rwanda; ^8^ Dataroots BV, Leuven, Belgium

**Keywords:** epilepsy, knowledge, attitude, practices, community health workers, training

There were three mistakes in “**Table 4**. Attitudes, knowledge and practices towards epilepsy. Survey on epilepsy Knowledge, Attitudes and Practices in Community Health Workers, Rwanda, 2018.” as published. Under the question “A child living with epilepsy should never attend school?”, it read “True (D)/False (UD)/Sometimes true (UD)/Not familiar (UD)”, with (D) = desired, (UD) = undesired. This has been corrected to “True (UD)/False (D)/Sometimes true (UD)/Not familiar (UD).”

Under the question “What is the cause of epilepsy” row “Genetic trait(D)” column “82 respondents” it read “21(13,0.5)”, this has been corrected to “21 (13.5)”.

Under the question “May your daughter marry a man living with epilepsy?” row “No (UD)” column “N=96” it read “12 (12,0.5)”, this has been corrected to “12 (12.5)”. The corrected “**Table 4**. Attitudes, knowledge and practices towards epilepsy. Survey on epilepsy Knowledge, Attitudes and Practices in Community Health Workers, Rwanda, 2018.” appears below.

The authors apologize for this error and state that it does not change the scientific conclusions of the article in any way. The first published incorrect version of the article has been updated.

**TABLE 4:** Attitudes, knowledge and practices towards epilepsy. Survey on epilepsy Knowledge, Attitudes and Practices in Community Health Workers, Rwanda, 2018.

	Group A (N = 96)	Group B (N = 103)
	n (%)	n (%)
KNOWLEDGE		
**Have you heard/read about epilepsy?**	N = 96	N = 103
Yes (D)	95 (99.0)	91 (88.3)
No (UD)	1 (1.0)	12 (11.7)
	*p* = 0.006	
**Which age group is most affected**?^a^	94 respondents	101 respondents
	N = 95	N = 109
0–4 (UD)	7 (7.4)	27 (24.8)
5–9 (UD)	21 (22.1)	34 (31.2)
≥10 (D)	67 (70.5)	48 (44.0)
	*p* < 0.001	
**What is the cause of epilepsy?** ^a^	95 respondents	82 respondents
	N = 206	N = 156
Brain injury (D)	86 (41,7)	61 (39.1)
Genetic trait (D)	14 (6.8)	21 (13.5)
Birth injury (D)	59 (28.6)	13 (8.3)
Excessive worry (UD)	27 (13.1)	15 (9.6)
Blood disorders (D)	17 (8.3)	17 (10.9)
Witchcraft (UD)	1 (0.5)	1 (0.8)
Curse of God (UD)	0 (0.0)	0 (0.0)
Spirit possession (UD)	1 (0.5)	8 (5.1)
Not familiar (UD)	1 (0.5)	20 (12.8)
	*p* < 0.001	
**Is epilepsy… ^a^**	96 respondents	89 respondents
	N = 114	N = 105
Madness (UD)	6 (5.3)	9 (7.6)
Spirit possession (UD)	12 (10.5)	13 (11.0)
Mental retardation (UD)	3 (2.6)	5 (4.2)
Brain disease (D)	93 (81.6)	78 (66.1)
Not familiar (UD)	0 (0.0)	13 (11.0)
	*p* = 0.013	
ATTITUDES		
**Would you allow your child to play with a child living with epilepsy?**	N = 96	N = 103
Yes (D)	93 (96.9)	84 (81.6)
No (UD)	3 (3.1)	7 (6.8)
Not familiar (UD)	0	12 (11.7)
	*p* = 0.003	
**A child living with epilepsy should never attend school?**	N = 96	N = 102
True (UD)	11 (11.5)	16 (15.7)
False (D)	80 (83.3)	70 (68.6)
Sometimes true (UD)	5 (5.2)	4 (3.9)
Not familiar (UD)	0 (0.0)	12 (11.8)
	*p* = 0.011	
**May your son marry a woman living with epilepsy?**	N = 95	N = 102
Yes (D)	80 (84.2)	51 (50.0)
No (UD)	15 (15.8)	38 (37.3)
Not familiar (UD)	0 (0.0)	13 (12.7)
	*p* < 0.001	
**May your daughter marry a man living with epilepsy?**	N = 96	N = 103
Yes (D)	84 (87.5)	52 (50.5)
No (UD)	12 (12.5)	40 (38.8)
Not familiar (UD)	0 (0.0)	11 (10.7)
	*p* < 0.001	
**Can you give a job to a person living with epilepsy?**	N = 96	N = 102
Yes (D)	91 (94.8)	68 (66.7)
No (UD)	5 (5.2)	34 (33.3)
Not familiar (UD)	0 (0.0)	0 (0.0)
	*p* < 0.001	
**Is epilepsy is contagious?**	N = 94	N = 100
Always (UD)	1 (1.1)	7 (7.0)
Sometimes (UD)	16 (17.0)	29 (29.0)
Never (D)	77 (81.9)	64 (64.0)
Not familiar (UD)	0 (0.0)	0 (0.0)
	*p* = 0.026	
PRACTICES		
**If a person found on the road what would you do?^a^**	96 respondents	97 respondents
	N = 96	N = 110
Health facility (D)	95 (99.0)	84 (76.4)
Traditional healer (UD)	0 (0.0)	3 (2.7)
Chinese medicine (UD)	0 (0.0)	3 (2.7)
Praying session (UD)	0 (0.0)	5 (4.5)
Do nothing as there is no treatment (UD)	0 (0.0)	5 (4.5)
Do not know (UD)	1 (1.0)	4 (3.6)
Not familiar (UD)	0 (0.0)	6 (5.5)
	*p* = 0.025	
**What would you recommend for treatment for a family member living with epilepsy?^a^**	96 respondents	98 respondents
	N = 106	N = 121
Health facility (D)	96 (90.6)	92 (76.0)
Traditional healer (UD)	0 (0.0)	8 (6.6)
Chinese medicine (UD)	2 (1.9)	3 (2.5)
Prayers (UD)	8 (7.5)	11 (9.1)
No, as epilepsy cannot be treated (UD)	0 (0.0)	2 (1.7)
Do not know what to do (UD)	0 (0.0)	1 (0.8)
Not familiar (UD)	0 (0.0)	4 (3.3)
	*p* = 0.18	
**How long should the treatment be given?^b^**	94 respondents	102 respondents
	N = 98	N = 102
For 1 week (UD)	3 (3.1)	11 (10.8)
For 1 year (UD)	5 (5.1)	7 (6.9)
For 2 years of seizure freedom (D)	45 (45.9)	17 (16.7)
For life (D)	45 (45.9)	67 (65.6)
	*p* < 0.001	

^a^ More than one answer was allowed.

^b^ Some Community Health Workers selected two answers with n, number responses; D, desired response; UD, undesired response.

